# Transposon-associated TnpB is a programmable RNA-guided DNA endonuclease

**DOI:** 10.1038/s41586-021-04058-1

**Published:** 2021-10-07

**Authors:** Tautvydas Karvelis, Gytis Druteika, Greta Bigelyte, Karolina Budre, Rimante Zedaveinyte, Arunas Silanskas, Darius Kazlauskas, Česlovas Venclovas, Virginijus Siksnys

**Affiliations:** Institute of Biotechnology, Life Sciences Center, Vilnius University, Vilnius, Lithuania

**Keywords:** DNA, Transposition

## Abstract

Transposition has a key role in reshaping genomes of all living organisms^1^. Insertion sequences of IS200/IS605 and IS607 families^2^ are among the simplest mobile genetic elements and contain only the genes that are required for their transposition and its regulation. These elements encode *tnpA* transposase, which is essential for mobilization, and often carry an accessory *tnpB* gene, which is dispensable for transposition. Although the role of TnpA in transposon mobilization of IS200/IS605 is well documented, the function of TnpB has remained largely unknown. It had been suggested that TnpB has a role in the regulation of transposition, although no mechanism for this has been established^3–5^. A bioinformatic analysis indicated that TnpB might be a predecessor of the CRISPR–Cas9/Cas12 nucleases^6–8^. However, no biochemical activities have been ascribed to TnpB. Here we show that TnpB of *Deinococcus radiodurans* ISDra2 is an RNA-directed nuclease that is guided by an RNA, derived from the right-end element of a transposon, to cleave DNA next to the 5′-TTGAT transposon-associated motif. We also show that TnpB could be reprogrammed to cleave DNA target sites in human cells. Together, this study expands our understanding of transposition mechanisms by highlighting the role of TnpB in transposition, experimentally confirms that TnpB is a functional progenitor of CRISPR–Cas nucleases and establishes TnpB as a prototype of a new system for genome editing.

## Abstract (web summary)

The RNA-directed nuclease TnpB from *Deinococcus radiodurans* can be reprogrammed to cleave DNA target sites in human cells.

## Main

**Fig. 1:**
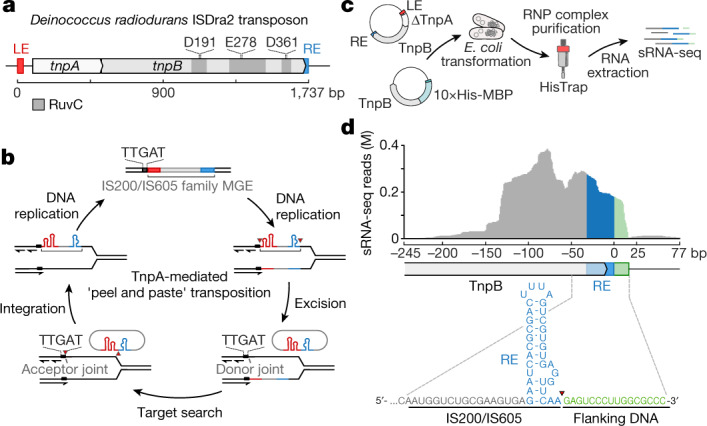
ISDra2 MGE of the IS200/IS605 family. **a**, Schematic representation of the *D. radiodurans* ISDra2 locus. The MGE consists of the *tnpA* and *tnpB* genes flanked by left end (LE) and right end (RE) partially palindromic elements (shown in red and blue, respectively). Amino acid residues at the predicted RuvC nuclease active site are indicated above the *tnpB* gene. **b**, TnpA-mediated ‘peel and paste’ transposition mechanism for ISDra2. The TnpA dimer catalyses transposon excision from the lagging strand during DNA replication forming a circular single-stranded DNA intermediate and a donor joint. The excised transposon circle inserts at the acceptor joint into the lagging DNA strand 3′ to the TTGAT sequence, completing the transposition cycle. Transposon excision/insertion sites are marked by red triangles. **c**, Experimental workflow of the expression and purification of the TnpB complex from *E. coli* cells and bound RNA extraction. sRNA-seq, small RNA sequencing. **d**, Alignment of sRNA sequenced reads to the ISDra2 locus. The blue colour shows the RNA sequences derived from the RE element, and the green marks the last 16 nt at the sequenced RNA 3′ ends, which align with the transposon flanking DNA.

Insertion sequences are widespread mobile genetic elements (MGEs) that only contain genes that are required for transposition and its regulation. Insertion sequences from the IS200/IS605 and IS607 families are among the simplest and most ancient MGEs^2^. Typically, they carry subterminal left end (LE) and right end (RE) palindromic elements at MGE ends and encode either *tnpA* and *tnpB* genes in various configurations, or isolated *tnpA* or *tnpB* genes (ISfinder database)^9^. The well-characterized *D. radiodurans* ISDra2 of the IS200/IS605 family consists of partially overlapping *tnpA* and *tnpB* genes flanked by LE and RE elements^10–12^ (Fig. 1a). The transposon mobilization occurs through a single-strand ‘peel and paste’ mechanism^13^ (Fig. 1b). ISDra2 TnpA, an extremely small (140 amino acids) Y1 transposase of the HUH family, excises a specific DNA strand near the 5′-TTGAT sequence to form a single-stranded transposon circle that is then integrated 3′ to the TTGAT target in a new location to complete the transposition cycle without duplication of the target site^10,12^. Another well-studied MGE, IS608 from *Helicobacter pylori*, follows the same mechanism but using a 5′-TTAC tetranucleotide sequence instead of the 5′-TTGAT pentanucleotide targeted by ISDra2 (refs.^4,14,15^). In both cases, excision or insertion of a single-stranded transposon circle occurs on the lagging DNA strand at the replication fork, coupling the transposition with the DNA replication cycle of the host^16^.

Although the function of TnpA in transposition is well established, the role of TnpB remains elusive. ISDra2 TnpB (408 amino acids) is not essential for transposition and is thought to regulate excision and insertion of transposons^3–5^, although a regulatory mechanism has yet to be established. Bioinformatic prediction of the conserved RuvC-like active site in the TnpB sequence led to speculations that TnpB could be an ancestor of Cas9 and Cas12 nucleases adopted by CRISPR–Cas systems^6–8^. However, neither the role of the RuvC motif in transposition nor the nuclease activity of TnpB have been experimentally demonstrated.

## TnpB forms an RNP complex with reRNA

**Extended Data Fig. 1:**
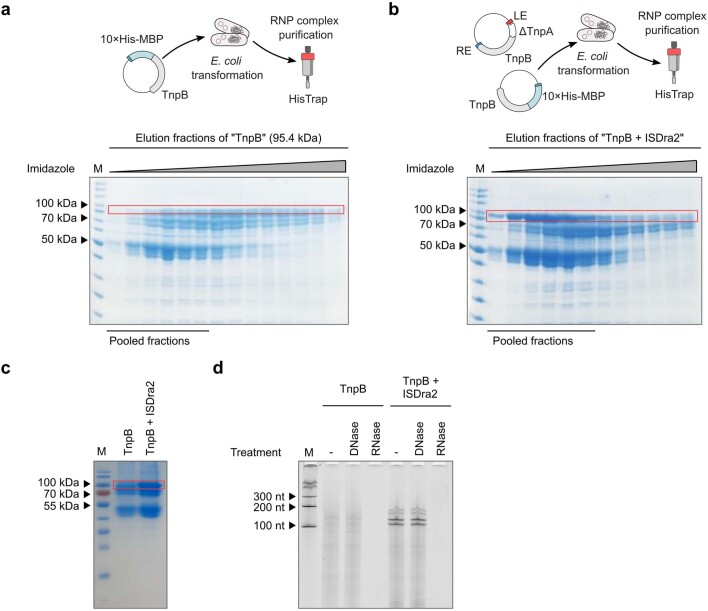
Purification of ISDra2 TnpB. **a**, **b**, Experimental workflow for expression and purification of a single TnpB (**a**) and TnpB co-expressed with ISDra2 (ΔTnpA) (**b**) in *E. coli* cells. SDS-PAGE gels show elution fractions of proteins bound to HisTrap chelating column. Red boxes denote bands corresponding to an intact 10×MBP-TnpB protein (95.4 kDa). **c**, SDS-PAGE gel of pooled fractions indicated in **a** and **b**. **d**, Detection and analysis of nucleic acids co-purifying with TnpB protein. For uncropped gel images, see Supplementary Fig. 2.

To establish the biochemical function of TnpB in the *D. radiodurans* ISDra2 element, we aimed to isolate and biochemically characterize the TnpB protein. Initially, we expressed *tnpB* fused to the sequence encoding the 10×His-maltose-binding protein (MBP) tag in *Escherichia coli* but failed to isolate the intact TnpB protein from cell extracts by Ni^2+^-affinity chromatography due to low yield (Extended Data Fig. 1a). However, co-expression of *tnpB* with a full ISDra2 transposon (with inactivated *tnpA*) resulted in a substantially increased TnpB yield, suggesting that additional transposon elements are required for stable expression of TnpB (Extended Data Fig. 1b, c). Subsequent biochemical analysis of TnpB samples revealed that RNA co-purified with the TnpB protein (Extended Data Fig. 1d). To characterize TnpB-bound RNAs, we performed small RNA sequencing that revealed the enrichment of non-coding RNAs approximately 150 nucleotides (nt) long derived from ISDra2 transposon RE element that we termed right end element RNAs (reRNAs) (Fig. 1c, d). The reRNA co-purified with TnpB fully matched to the 3′ end of the *tnpB* gene and RE sequence, except for the last approximately 16 nt at the 3′ end, which were derived from the DNA sequence flanking the IS200/IS605 transposon (Fig. 1d). The enrichment of non-coding RNAs associated with *tnpB*-encoding transposons from the IS200/IS605 family has been previously reported; however, their function remains elusive^17,18^. Here we provide experimental evidence that TnpB forms a ribonucleoprotein (RNP) complex with reRNA derived from the 3′ end of the transposon .

## TnpB RNP cleaves DNA in vitro

**Fig. 2:**
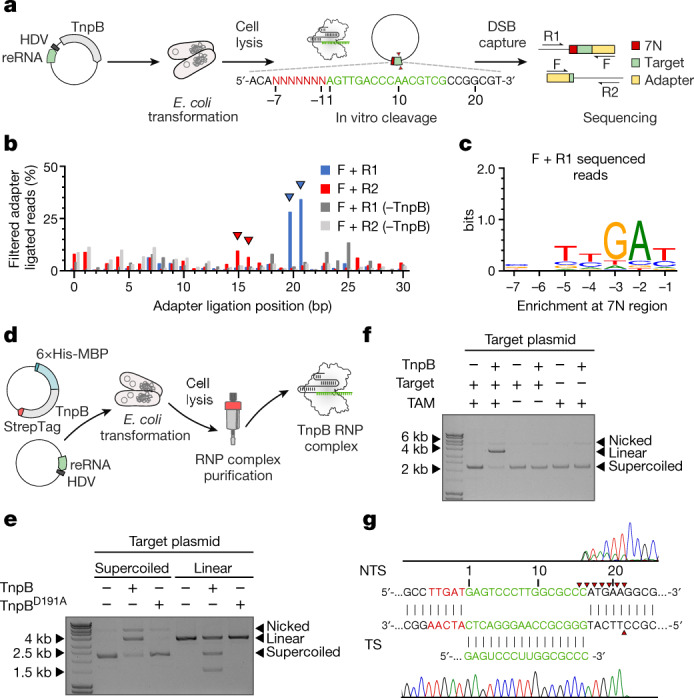
TnpB protein is an RNA-guided dsDNA nuclease. **a**, Experimental workflow for the establishment of dsDNA cleavage requirements by the TnpB–reRNA complex. *E. coli* cells were transformed with a plasmid expressing TnpB and HDV ribozyme-terminated reRNA, containing the 16-nt sequence that matched the target in the plasmid DNA library, flanked by the randomized 7-nt sequence (7N). Cell lysate was used for library digestion followed by double-stranded break (DSB) capture. F, forward primer, annealing to the ligated adapter; R1 and R2, reverse primers, annealing to the target plasmid backbone. **b**, Determination of adapter ligation positions indicate the formation of DSBs in the targeted sequence. ‘–TnpB’ represents the cleavage reactions using lysates obtained from the cells that do not express TnpB. The blue and red triangles indicate the positions of F + R1-enriched and F + R2-enriched adapter ligated reads, respectively. **c**, WebLogo representation of motifs identified in the 7N randomized region at 20–21-bp F + R1-enriched adapter ligated reads. **d**, Experimental workflow of the expression and purification of the TnpB RNP complex. *E. coli* cells were transformed with a plasmid expressing TnpB and a separate plasmid expressing HDV ribozyme-terminated reRNA. The reRNA-encoding construct contained the 16-nt guide sequence, which was different from the guide sequence used in the plasmid library cleavage experiment. **e**, The TnpB RNP complex cleaves supercoiled and linearized target plasmid in vitro. Cleavage is blocked by the D191A mutation at the RuvC-like active site. **f**, Target plasmid cleavage (TAM+/Target+, TAM−/Target+ and TAM+/Target−) by the TnpB RNP complex in vitro. TAM and the target complementary to the reRNA 3′-end sequence are required for plasmid DNA cleavage. **g**, Sanger sequencing of the TnpB-cleaved plasmid products reveals multiple cleavage positions at the non-targeted strand (NTS) and a single cleavage site at the target strand (TS) (marked with red triangles). For uncropped gel images, see Supplementary Fig. 1.

**Extended Data Fig. 2:**
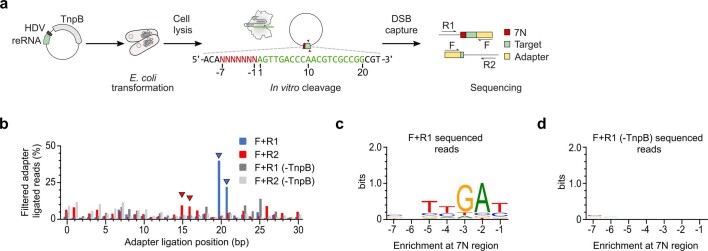
TnpB RNP complex cleaves dsDNA in a TAM dependent manner. **a**, Experimental workflow of double-stranded (ds) DNA cleavage activity detection. The reRNA encoding construct contained 20 nt guide sequence. F – forward primer, annealing to ligated adapter. R1 and R2 – reverse primers, annealing to the plasmid backbone. 7N represents the randomized region in the plasmid library next to the targeted sequence. **b**, Adapter ligation position determination indicating double-stranded break (DSB) formation in the targeted sequence. “–TnpB” represents the cleavage reactions using lysates obtained from the cells that do not express TnpB. **c**, **d**, WebLogo representation of motifs identified in 7N randomized region at 20–21 bp F + R1 and F + R1 (-TnpB) enriched adapter ligated reads, respectively. No substantial enrichment is observed at 7N region for “-TnpB” reactions.

**Extended Data Fig. 3:**
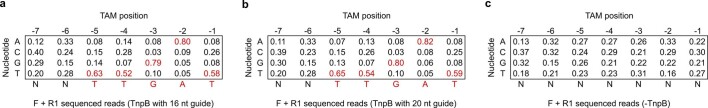
Position frequency matrix (PFM) of the nucleotides at 7N region obtained after plasmid library cleavage using *E. coli* lysates. **a**, **b**, PFM representation of nucleotides distribution identified in 7N randomized region at 20–21 bp F + R1 enriched adapter ligated reads obtained from *E. coli* cells expressing TnpB and reRNA constructs with 16 nt and 20 nt guide sequences, respectively. **c**, the cleavage reaction using lysate obtained from the cells that do not express TnpB demonstrates no substantial enrichment of motifs at 7N region.

Guided by the similarities of TnpB to the CRISPR–Cas12f effector complexes that function as RNA-guided double-stranded DNA (dsDNA) nucleases^19^, we hypothesized that the approximately 16-nt 3′ terminal of reRNA, which are derived from the DNA adjacent to the transposon and would be variable per se (Fig. 1d), might function as a guide sequence that directs TnpB to its target and activates DNA cleavage. To test this hypothesis, we adopted the previously developed protospacer adjacent motif (PAM) identification assay for Cas9/Cas12 nucleases^19^. First, we engineered the plasmids encoding TnpB and reRNA, where the 3′-terminal reRNA 16-nt sequence was replaced by 16-nt (Fig. 2a) or 20-nt (Extended Data Fig. 2a) sequences that matched the target next to the 7-nt (7N) randomized region in the plasmid library. Next, following *E. coli* transformation and expression, cell lysates containing TnpB RNP complexes were used for plasmid library cleavage. The DNA ends that would result from the plasmid cleavage were repaired by T4 DNA polymerase, subjected to adapter ligation, PCR amplified and sequenced. Sequencing of the adapter-ligated fragments revealed enrichment of the products having adapters at the target site 20–21 bp (targeted strand) and 15 bp (non-targeted strand) from the randomized region, indicating cleavage of the plasmid library by the TnpB RNP complex (Fig. 2b, Extended Data Fig. 2b). The positions of the adapter ligation for the targeted strand and non-targeted strand suggested a staggered cleavage pattern generating 5′ overhangs. Further analysis of DNA fragments revealed enrichment of TTGAT sequences in the randomized 7N region 5′ upstream of the target sequence (Fig. 2c, Extended Data Figs. 2c, d, 3). Notably, the TTGAT sequence that licensed cleavage of the plasmid library by TnpB matched the target site sequence that is required for TnpA-mediated ISDra2 transposon excision and insertion^11^. Because this sequence was equivalent to the PAM sequence that is required for initiation of DNA cleavage by Cas9 or Cas12 nucleases, we termed it transposon-associated motif (TAM).

**Extended Data Fig. 4:**
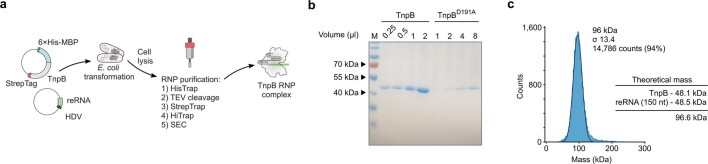
TnpB RNP complex purification. **a**, Experimental workflow of TnpB RNP complex expression and multi-step purification. The reRNA encoding construct contained a 16 nt guide sequence and carried an HDV ribozyme sequence at the 3’-end, which enabled production of the reRNA with fixed 16 nt length guide sequence. **b**, SDS-PAGE analysis of the purified TnpB and TnpB (D191A) RNP complexes. For uncropped gel image, see Supplementary Fig. 2. **c**, Molecular mass of TnpB and reRNA RNP complex determined by mass photometry. Experimentally established molecular mass corresponds to TnpB RNP complex consisting of a TnpB protein monomer bound to a ~150 nt long reRNA in a 1:1 molar ratio.

**Extended Data Fig. 5:**
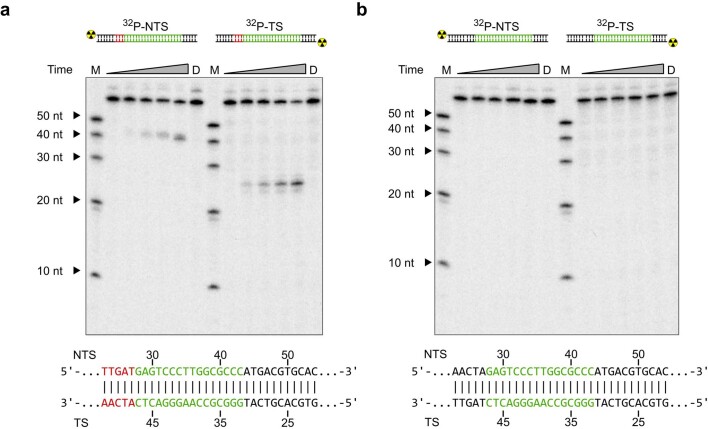
Synthetic dsDNA cleavage by TnpB RNP complex. **a**, **b**, Cleavage of dsDNA substrates containing a target (represented in green colour) with TAM (red colour) (**a**) or without TAM sequence (**b**). TnpB RNP complex cleaves dsDNA in a TAM-dependent manner. Cleavage is blocked by D191A mutation at the RuvC-like active site (lane D). NTS and TS represent non-target and target strand, respectively. M – DNA marker lane. For uncropped gel images, see Supplementary Fig. 2.

**Extended Data Fig. 6:**
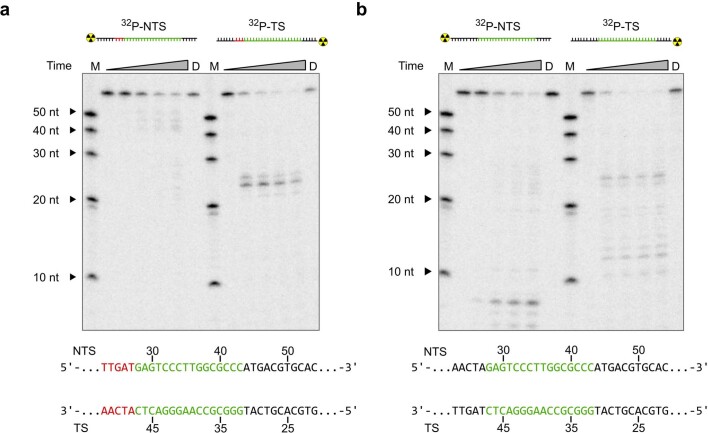
Synthetic ssDNA cleavage by TnpB RNP complex. **a**, **b**, Cleavage of ssDNA substrates containing a target (represented in green colour) with TAM (red colour) (**a**) or without TAM sequence (**b**). TnpB RNP complex cleaves ssDNA in a TAM-independent manner. Cleavage is blocked by D191A mutation at the RuvC-like active site (lane D). NTS and TS represent non-target and target strand, respectively. M – DNA marker lane. For uncropped gel images, see Supplementary Fig. 2.

Next, to validate the requirements for dsDNA cleavage established on the plasmid DNA library, we purified the TnpB–reRNA complex with 1:1 stoichiometry (Fig. 2d, Extended Data Fig. 4) and tested its ability to cleave dsDNA substrates (Fig. 2e, f, Extended Data Fig. 5). First, incubation of the TnpB complex with the plasmid (both supercoiled and linearized), containing the target next to the TAM sequence, converted the supercoiled plasmid into its linear form or yielded linear DNA cleavage products of expected size, respectively (Fig. 2e). Mutation of the conserved D191 residue in the RuvC-like active site compromised cleavage, indicating that the RuvC motif is responsible for cleavage of dsDNA (Fig. 2e). DNA cleavage required both TAM and the target-matching sequence at the 3′ end of reRNA (Fig. 2f). Last, run-off sequencing of the cleavage products confirmed a staggered cleavage pattern at 15–21 bp from the TAM that resulted in 5′ overhangs (Fig. 2g). Data for the synthetic oligonucleotide cleavage (Extended Data Fig. 5) were consistent with the plasmid DNA cleavage experiments (Fig. 2e, f). TnpB RNP also cleaved a matching single-stranded DNA in a TAM-independent manner (Extended Data Fig. 6). Together, these results demonstrate that TnpB functions as a TAM-dependent RNA-guided dsDNA nuclease.

## TnpB ensures DNA interference in *E. coli*

**Fig. 3:**
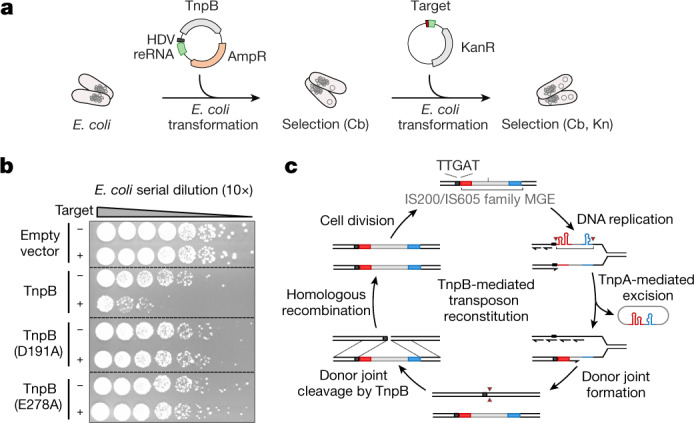
TnpB-mediated plasmid interference in vivo. **a**, Experimental workflow of the plasmid interference assay in *E. coli*. The cleavage of a target plasmid results in loss of kanamycin (Kn) resistance. The reRNA-encoding construct contained the 16-nt guide sequence. AmpR, ampicillin/carbenicillin (Ap/Cb) resistance gene; KanR, kanamycin resistance gene. **b**, Plasmid interference assay. *E. coli* culture samples were serially diluted (10×) and the *E. coli* transformants were grown on the media supplemented with Cb and Kn at 25 °C for 44 h. Interference is compromised for the catalytically dead D191A and E278A TnpB variants. Target ‘+’ or ‘−’ indicates the plasmids with or without the target, respectively. For the uncropped plate image, see Supplementary Fig. 1. **c**, Proposed role of TnpB in transposition. The IS200/IS605 transposon circle is excised from the lagging strand during DNA replication resulting in two DNA copies: one copy that originates from the leading strand and carries an intact transposon, and another copy that originates from the lagging strand and lacks the transposon at the original site due to the strand-specific transposon excision. However, the latter DNA copy still carries the transposon ‘footprint’ in the form of the donor joint, comprised of the 5′-TTGAT sequence and the 3′-flanking DNA sequence that becomes a target to the TnpB–reRNA complex. In this case, the 5′-TTGAT sequence serves as a TAM that initiates the binding of the reRNA sequence to the matching DNA sequence followed by dsDNA cleavage. TnpB-induced DSB could facilitate homology-directed repair to reinstate the transposon at the donor joint using its intact copy on the sister chromatid, ensuring that both DNA copies have a transposon-coding gene before cell division. Red triangles indicate DNA cleavage sites.

To probe whether TnpB is able to cleave its target in the cells, we adopted a plasmid interference assay (Fig. 3a). In brief, *E. coli* cells expressing the TnpB complex were transformed with a plasmid containing the TAM-flanked target and carrying the kanamycin resistance gene that enable growth on kanamycin-supplemented agar plates (Fig. 3a). Serial dilutions of the transformants revealed plasmid interference in the cells containing the native TnpB variant but not TnpB with the mutated RuvC motif (Fig. 3b). Together, these results confirm that TnpB can cleave dsDNA target in vivo. The dsDNA cleavage activity of TnpB and the target site requirements established here allow us to propose the role of TnpB in the transposition of IS200/IS605 MGEs (Fig. 3c). We suggest that TnpB guided by reRNA makes a double-stranded break (DSB) at the 5′-TTGAT flanked donor joint site that is formed in DNA when the transposon circle is excised during replication. A TnpB-induced DSB could then facilitate homology-directed repair to reinstate the transposon at the donor joint using its intact copy on the sister chromatid, ensuring that both DNA copies secured a transposon copy before cell division (Fig. 3c).

## TnpB cleaves genomic DNA in human cells

**Fig. 4:**
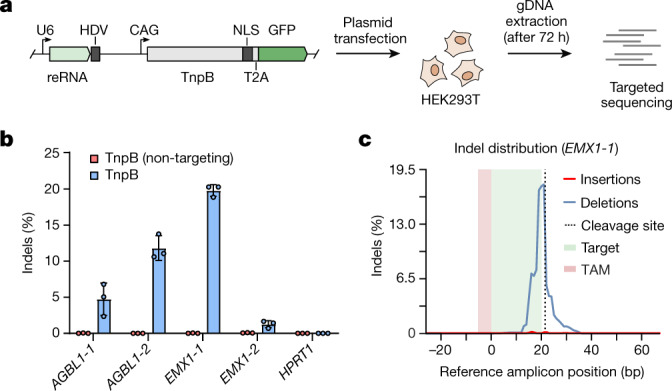
TnpB nuclease is a novel genome editor. **a**, The experimental workflow of the human cell line (HEK293T) genome-editing experiment. NLS, nuclear localization sequence. **b**, Detection of indel activity in the five tested targets of 20 nt in length in human gDNA (represented as the mean of three biologically independent experiments (shown in dots) ± standard deviation). The TnpB (non-targeting) expression plasmid used as a negative control encodes the reRNA-containing guide sequence that does not match any target in the human gDNA. **c**, Indel profile distribution within the target sequence in the *EMX1-1* site showing the distribution of deletions (blue line) and insertions (red line) across the cleavage site (dotted line). The profile was obtained by aligning all reads at the *EMX1-1* site and counting deletions and insertions at each position.

Finally, after demonstrating RNA-guided dsDNA cleavage both in vitro and in *E. coli*, we tested whether TnpB can be adopted for genome editing of human cells (HEK293T). Plasmids encoding the TnpB protein fused with a nuclear localization sequence and reRNA constructs targeting five 20-nt sites next to the 5′-TTGAT TAM sequence in human genomic DNA (gDNA) were transiently transfected into HEK293T cells (Fig. 4a). After 72 h, gDNA was extracted and analysed by sequencing for the presence of insertions and deletions (indels) at the targeted cleavage sites, indicating DSB repair events and genome editing. At the two tested sites (*AGBL1-2* and *EMX1-1*), TnpB introduced mutations at frequencies of 10–20% (Fig. 4b), similar to the levels observed for CRISPR–Cas9 and Cas12-based editing^20–25^. *AGBL1-1* and *EMX1-2* sites were moderately (1–5%) modified, whereas no indels were detected at the *HPRT1* site. Further analysis of the obtained indels revealed a domination of deletions at the cleavage site over insertions (Fig. 4c), similar to the mutational profiles observed after Cas12 cleavage^23,25^. Therefore, these results indicate that compact RNA-guided TnpB nucleases can cleave eukaryotic gDNA and may be adopted as tools for genome editing.

## Discussion

Overall, in this study, we identified the functional activity of the TnpB protein from the ISDra2 system by demonstrating TAM-dependent RNA-guided dsDNA cleavage. This observation expands our understanding of the transposition mechanisms of MGEs in the large IS200/IS605 family. Although TnpA has been widely studied, revealing the TnpA-mediated ‘peel and paste’ transposition mechanism, TnpB is not essential for transposition but may have a role in its regulation as it has been suggested for ISDra2 TnpB, although no mechanism for this has been established^5^. The inhibitory effect of TnpB on the excision and integration steps of ISDra2 transposition reported previously^5^ should be revisited, taking into consideration the RNA-guided TnpB nuclease activity demonstrated in this work.

Here we show that both in vitro (Fig. 2e, f) and in *E. coli* cells (Fig. 3b), TnpB cleaves the donor joint that would be generated after transposon excision and propose that TnpB-mediated DSB triggers homology-directed repair to reinstate the transposon into its original site. This process would be analogous to the group I intron homing promoted by intron encoded endonucleases^26^. We propose that in MGEs containing both *tnpA* and *tnpB*, two types of transposition will occur: (1) excision of the transposon and insertion of it at a new site (catalysed by TnpA) (Fig. 1b); and (2) transposon ‘homing’, a process in which TnpB cuts DNA in a transposon-less allele, triggering recombination that copies the transposon into the same position (Fig. 3c). TnpB thus provides a backup mechanism that prevents the possible loss of MGEs if the integration step of the excised transposon is unsuccessful and ensures that both daughter cells will acquire identical DNA copies. In this case, the ‘peel and paste’ mechanism, which was proposed for TnpA, in the presence of TnpB is transformed into a ‘peel, paste and copy’ mechanism. The RNA-guided DNA cleavage activity of TnpB could also enable transposon integration into ectopic sites flanked by homologous sequences containing the TAM and matching guide RNA, thus providing an additional mechanism for transposon propagation.

**Extended Data Fig. 7:**
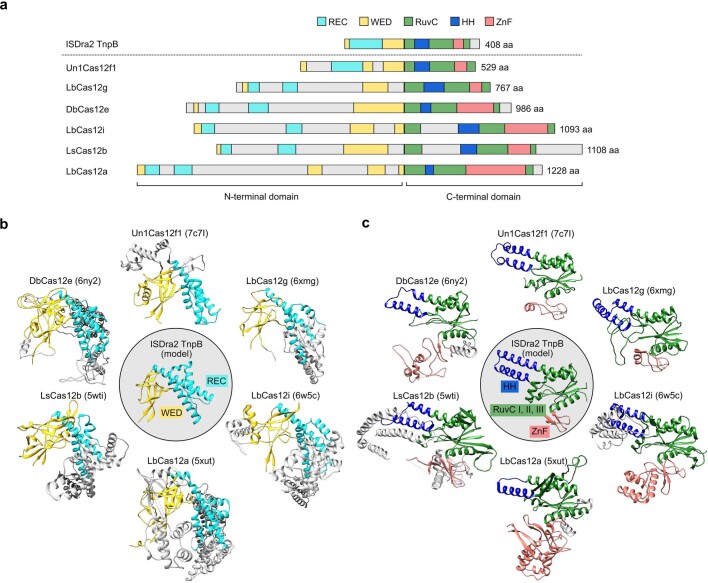
Comparison of a TnpB model with experimentally determined representative structures of different Cas12 groups. **a**, Schematic representation of common structural domains/motifs (coloured) and unique structural regions (grey) along the sequence; WED – wedge domain, corresponding to the β-barrel, REC – helical bundle, RuvC – RuvC domain with the inserted helical hairpin (HH) and either the zinc-finger domain (ZnF), or analogous domain inserted in the same relative position. Length of all sequences are approximately to scale. TnpB represents a minimal domain organization also present in Cas12 groups. **b**, Comparison of N-terminal regions between TnpB and other Cas12 proteins; the N-terminal WED and REC domains are involved in PAM recognition in Cas12f and are expected to participate in TAM recognition in TnpB. **c**, Comparison of corresponding RuvC regions.

Recently, the evolution of CRISPR–Cas9 nucleases from IscB proteins of the distinct IS200/IS605 transposon family has been reconstructed and RNA-guided DNA cleavage activity of the TnpB protein has been reported; however, the role of TnpB in transposition has not been discussed in detail^27^. The demonstration of the RNA-guided dsDNA cleavage activity of TnpB provides a direct experimental confirmation of an evolutionary scenario for class 2 CRISPR–Cas systems, pinpointing MGEs as predecessors of Cas9 and Cas12 effectors^6–8^. Sequence comparisons of TnpB and Cas12 family proteins show similar domain organizations, including a conserved RuvC endonuclease-like motif (Extended Data Fig. 7). The closest TnpB neighbours on the evolutionary tree are the miniature Cas12f nucleases^8,19,28^. However, there are important differences between TnpB and the Cas12f nucleases. First, whereas Cas12f nucleases use a guide RNA that originates from the CRISPR array, TnpB uses right transposon element-derived reRNA as a guide. Next, TnpB is a monomer and requires a single reRNA molecule (Extended Data Fig. 3c), whereas Cas12f nucleases are dimers that bind to a single copy of a crRNA (CRISPR RNA)–tracrRNA (trans-activating crRNA) duplex^29,30^. Last, although the TAM sequence required for TnpB cleavage seems to be equivalent to the PAM sequence that licenses Cas12f cleavage, Cas12f proteins show distinct PAM requirements^19^. It would be interesting to see whether the PAM diversity of Cas12f nucleases correlate with the distinct TAM sequence requirements of TnpB proteins, reflecting evolutionary relationships between Cas12 nucleases and MGEs of the IS200/IS605/IS607 families. RNA-guided DNA insertion by CRISPR-associated Tn7-like transposons provides another example of the interplay between MGEs and CRISPR–Cas systems^31,32^.

Finally, we show that TnpB also cleaves dsDNA in human cells and expands the genome-editing toolbox by providing a new class of extremely compact non-Cas nucleases with different biochemical requirements for genome-editing applications (Extended Data Table 1). The natural diversity of TnpB orthologues, including eukaryotic variants that remain to be characterized^33^, and their miniature size suitable for adeno-associated virus-based delivery open new horizons for human therapeutic applications.

## Methods

### Engineering of TnpB expression vectors

The IS200/IS605 ISDra2 system from *D. radiodurans* R1 (GenBank AE000513.1) was ordered as a synthetic sequence cloned into the pTwist vector under the T7 promoter (pTWIST-ISDra2; Twist Biosciences). To obtain the ISDra2 variant with a deletion within the *tnpA* gene (pGD3), the pTWIST-ISDra2 plasmid was pre-cleaved with NdeI (Thermo Fisher Scientific), 5′ overhangs filled-in using T4 DNA polymerase (Thermo Fisher Scientific) and self-circularized with T4 DNA ligase (Thermo Fisher Scientific). For TnpB purification, two pBAD-derived expression vectors were constructed: the *tnpB*-encoding sequence was fused to N-terminal 10×His-TwinStrep-MBP (pTK120-ISDra2-TnpB) or N-terminal 6×His-MBP and C-terminal StrepTag II (pTK151) protein purification tags and cloned under arabinose-inducible promoters using the NEBuilder HiFi DNA Assembly Kit (New England Biolabs). To obtain the reRNA expression vector (pGB71) for TnpB RNP complex purification, the reRNA-encoding sequence with the T7 promoter at the 5′ end and hepatitis delta virus (HDV) ribozyme with the T7 terminator at the 3′ end (assembled by PCR from synthetic oligonucleotides) was cloned into the pACYC184 vector between HindIII and BclI restriction sites (Thermo Fisher Scientific). The self-cleaving HDV ribozyme ensured fixed 16-nt guide RNA length at the 3′ end of reRNA. pGB74-78, containing reRNA-encoding and *tnpB*-encoding sequences under T7 and T7lac promoters, respectively, used for TnpB complex expression in 7N plasmid library cleavage and plasmid interference assays, were obtained by cloning the reRNA-encoding construct between Bsu15I and EcoRI, and *tnpB* between NdeI and XhoI (Thermo Fisher Scientific) restriction sites into the pETDuet-1 vector (Novagen). For genome-editing experiments in human HEK293T cells, reRNA (targeting 20-bp sites in human gDNA) and TnpB (fused at the 3′ end with SV40 nuclear localization sequence (NLS)-T2A-GFP) encoding constructs were cloned into the pX458-derived plasmid (a gift from F. Zhang, Addgene plasmid #48138) under U6 and CAG promoters, respectively (pRZ122-127), using the NEBuilder HiFi DNA Assembly Kit (New England Biolabs). The Phusion Site-Directed Mutagenesis Kit (Thermo Fisher Scientific) was used to obtain plasmid variants with a mutated RuvC active site. The description of the TnpB expression plasmids and links to the sequences are provided in Supplementary Table 1.

### Expression and purification of the TnpB RNP complex

For the initial TnpB protein expression and purification, *E. coli* BL21-AI cells were transformed with pTK120-ISDra2-TnpB or pTK120-ISDra2-TnpB with pGD3 (plasmid encoding ISDra2 transposon with a deletion within the *tnpA* gene) and grown in LB medium, supplemented with ampicillin (100 µg/ml) or ampicillin (100 µg/ml) and chloramphenicol (50 µg/ml), respectively, at 37 °C. After culturing to an OD_600_ of 0.6–0.8, protein expression was induced with 0.2% arabinose and the cells were grown for an additional 16 h at 16 °C. Next, the cells were pelleted by centrifugation, resuspended in 20 mM Tris-HCl (pH 8.0 at 25 °C), 250 mM NaCl, 5 mM 2-mercaptoethanol, 25 mM imidazole, 2 mM PMSF and 5% (v/v) glycerol containing buffer and disrupted by sonication. After removing cell debris by centrifugation, the supernatant was loaded onto the Ni^2+^-charged HiTrap chelating HP column (GE Healthcare) and proteins were eluted with a linear gradient of increasing imidazole concentration from 25 mM to 500 mM in 20 mM Tris-HCl (pH 8.0 at 25 °C), 500 mM NaCl, 5 mM 2-mercaptoethanol and 5% (v/v) glycerol buffer. The fractions containing TnpB were pooled, dialysed against 20 mM Tris-HCl (pH 8.0 at 25 °C), 250 mM NaCl, 2 mM DTT and 50% (v/v) glycerol-containing buffer and stored at −20 °C. The obtained purified TnpB samples were used for nucleic acid extraction and analysis.

For increased expression and yield of the TnpB RNP complex, *E. coli* BL21-AI cells were transformed with reRNA (pGB71) and TnpB (pTK151) or TnpB^D191A^ (pTK152) expression vectors and grown in LB medium, supplemented with ampicillin (100 µg/ml) and chloramphenicol (50 µg/ml) at 37 °C. After culturing to an OD_600_ of 0.6–0.8, protein expression was induced with 0.2% arabinose and cells were grown for additional 16 h at 16 °C. Next, the cells were pelleted by centrifugation, resuspended in 20 mM Tris-HCl (pH 8.0 at 25 °C), 500 mM NaCl, 5 mM 2-mercaptoethanol, 25 mM imidazole, 2 mM PMSF and 5% (v/v) glycerol-containing buffer and disrupted by sonication. The supernatant obtained after centrifugation was loaded onto the Ni^2+^-charged HiTrap chelating HP column (GE Healthcare) and bound proteins were eluted with a linear gradient of increasing imidazole concentration from 25 mM to 500 mM in 20 mM Tris-HCl (pH 8.0 at 25 °C), 500 mM NaCl, 5 mM 2-mercaptoethanol and 5% (v/v) glycerol buffer. The fractions containing TnpB RNP complexes were pooled and the 6×His-MBP tag was cleaved by overnight incubation with TEV protease at 8 °C. Next, the reaction mixture was loaded onto the StrepTrap column (GE Healthcare), washed with 20 mM Tris-HCl (pH 8.0 at 25 °C), 150 mM NaCl, 5 mM 2-mercaptoethanol and 5% (v/v) glycerol buffer and bound TnpB complex eluted with 2.5 mM *d*-desthiobiotin solution. Fractions containing TnpB RNP were pooled, loaded on a HiTrap heparin HP column (GE Healthcare) and eluted using a linear gradient of increasing NaCl concentration from 0.15 M to 1.0 M. Obtained TnpB RNP complex fractions were pooled, concentrated up to 0.5 ml using the Amicon Ultra-15 centrifugal filter unit (Merck Millipore) and loaded on a Superdex 200 10/300 GL (GE Healthcare) gel filtration column equilibrated with 20 mM Tris-HCl (pH 8.0 at 25 °C), 250 mM NaCl and 5 mM 2-mercaptoethanol buffer. Peak fractions containing TnpB RNP complexes were pooled and dialysed against 20 mM Tris-HCl (pH 8.0 at 25 °C), 250 mM NaCl, 2 mM DTT and 50% (v/v) glycerol-containing buffer and stored at −20 °C. The concentration of the TnpB RNP complex was determined by quantifying the intensity of protein bands in SDS–PAGE gels and comparing them to the protein standard of known concentration. The sequences of TnpB protein constructs are listed in Supplementary Table 2.

### Molecular mass measurements by mass photometry

Measurement coverslips (no. 1.5 H, 24 × 50 mm; Marienfeld) were cleaned by sequential sonication for 5 min in MilliQ water, isopropanol and MilliQ water and then dried using a clean stream of nitrogen gas. A prepared coverslip was mounted onto the OneMP mass photometer (Refeyn) and a CultureWell Reusable Gasket (Grace Bio-Labs) was placed on top. A gasket well was filled with 10 µl of 20 mM Tris-HCl (pH 8.0 at 25 °C) and 250 mM NaCl buffer, 10 µl of the diluted TnpB RNP complex sample (approximately 60 nM) was added and the adsorption of biomolecules was monitored for 120 s using the AcquireMP software (Refeyn). For converting the measured ratiometric contrast into molecular mass, Un1Cas12f1 protein^19^ and its oligomers ranging from 60 kDa to 250 kDa (monomer to tetramer) were used for calibration. Samples were measured in triplicates. Mass photometry movies were analysed using DiscoverMP (Refeyn).

### Extraction and analysis of TnpB-bound nucleic acids

To extract TnpB-bound nucleic acids, 100 μl of purified TnpB samples was incubated with 5 μl (20 mg/ml) of proteinase K (Thermo Fisher Scientific) for 45 min at 37 °C in 1 ml of 10 mM Tris-HCl (pH 7.5 at 37 °C), 5 mM MgCl_2_, 100 mM NaCl, 1 mM DTT and 1 mM EDTA reaction buffer. Next, the mixtures were treated with phenol:chloroform:isoamyl alcohol (25:24:1) solution and the aqueous phase was subsequently mixed with chloroform to remove any remaining phenol. The solution with nucleic acids was split into fresh tubes (198 μl into each) and incubated with 2 µl of RNase I (10 U/μl) (Thermo Fisher Scientific) or DNase I (10 U/μl) (Thermo Fisher Scientific) for 45 min at 37 °C. Reaction products were mixed with 2× RNA loading dye (Thermo Fisher Scientific), separated on TBE-Urea (8 M) 15% denaturing polyacrylamide gel using 0.5× TBE electrophoresis buffer (Thermo Fisher Scientific) and visualized with SYBR Gold (Thermo Fisher Scientific).

### RNA isolation from the TnpB RNP complex

For TnpB-bound RNA extraction, 100 μl of purified TnpB complex was incubated with 5 μl (20 mg/ml) of proteinase K (Thermo Fisher Scientific) for 45 min at 37 °C in 1 ml of 10 mM Tris-HCl (pH 7.5 at 37 °C), 5 mM MgCl_2_, 100 mM NaCl, 1 mM DTT and 1 mM EDTA reaction buffer. DNA was removed by adding 10 μl of DNase I (10 U/μl) (Thermo Fisher Scientific) and incubating for an additional 45 min at 37 °C, and the reaction mixture was purified with a GeneJET RNA Cleanup and Concentration Micro Kit (Thermo Fisher Scientific). Next, 3 μg of purified RNAs was phosphorylated at 37 °C for 30 min using 1 μl (10 U/μl) of PNK (Thermo Fisher Scientific) in 1× reaction buffer A (Thermo Fisher Scientific) supplemented with 1 mM ATP (20 µl reaction volume). Reaction products were purified using a GeneJET RNA Cleanup and Concentration Micro Kit (Thermo Fisher Scientific).

### RNA sequencing and analysis

RNA libraries were prepared using Collibri Stranded RNA Library Prep Kit for Illumina Systems (Thermo Fisher Scientific) according to the manufacturer’s instructions for small RNAs (protocol MAN0025359), pooled in an equimolar ratio and pair-end sequenced (2 × 75 bp) using MiSeq Reagent Kit v2, 300 cycles (Illumina) on a MiSeq System (Illumina). The pair-end reads shorter than 20 bp were filtered with Cutadapt^34^. The remaining reads were mapped to the transposon-encoding plasmid (pTWIST-ISDra2; Supplementary Table 1) using BWA^35^ and converted to the BAM file format with SAMtools^36^. The resulting coverage data were visualized using Integrative Genomics Viewer^37^.

### TnpB cleavage of plasmid DNA library

For dsDNA cleavage detection and TAM characterization, the previously developed PAM determination assay for Cas9 and Cas12 effectors was adopted^19,38,39^. In brief, the *tnpB* gene and reRNA constructs, targeting 16-bp or 20-bp sequences in the plasmid library pTZ57 (Supplementary Table 1), adjacent to a 7N randomized region (Supplementary Table 3), were cloned into a pETDuet-1 (Millipore Sigma) vector (pGB77-78; Supplementary Table 1). Next, *E. coli* ArcticExpress (DE3) cells were transformed with TnpB RNP components encoding plasmids and the cells were grown in LB medium supplemented with ampicillin (100 μg/ml) and gentamicin (10 µg/ml). After reaching an OD_600_ of 0.5, TnpB expression was induced with 0.5 mM IPTG and the culture was incubated overnight at 16 °C. The cells from 10 ml of overnight culture were collected by centrifugation, resuspended in 1 ml of lysis buffer (20 mM phosphate (pH 7.0), 0.5 M NaCl, 5% (v/v) glycerol and 2 mM PMSF) and lysed by sonication. Cell debris was removed by centrifugation, and 10 μl of the obtained TnpB RNPs containing supernatant were used directly in the digestion experiments. In brief, lysate was mixed with 1 μg of the 7N randomized plasmid library (approximately 20 M fold coverage of all 7N TAM sequences) in 100 μl of reaction buffer (10 mM Tris-HCl (pH 7.5 at 37 °C), 100 mM NaCl, 1 mM DTT and 10 mM MgCl_2_) and incubated for 1 h at 37 °C. Cleaved DNA ends were repaired by adding 1 μl of T4 DNA polymerase (Thermo Fisher Scientific), 1 μl of 10 mM dNTP mix (Thermo Fisher Scientific) and incubating at 11 °C for 20 min, followed by heating the mix up to 75 °C for 10 min. Next, 3′-dA overhangs were added by incubating the reaction mixture with 1 μl of DreamTaq polymerase (Thermo Fisher Scientific) and 1 μl of 10 mM dATP (Thermo Fisher Scientific) for 30 min at 72 °C. In addition, RNA was removed by incubating for 15 min at 37 °C with 1 μl of RNase A (Thermo Fisher Scientific) and the DNA was purified using the GeneJet PCR Purification Kit (Thermo Fisher Scientific). The purified cleavage products (100 ng) were ligated with a dsDNA adapter containing a 3′-dT overhang (100 ng) for 1 h at 22 °C using 1 μl of T4 DNA ligase (Thermo Fisher Scientific) in 20 μl reaction volume. Next, the adapter-bearing cleavage products were PCR amplified and gel purified using the GeneJet Gel Purification Kit (Thermo Fisher Scientific). DNA libraries were prepared using the Collibri PS DNA Library Prep Kit for Illumina Systems (Thermo Fisher Scientific) according to the manufacturer’s instructions, pooled in an equimolar ratio and pair-end sequenced (2 × 150 bp) using MiSeq Reagent Kit v2, 300 cycles (Illumina) on a MiSeq System (Illumina).

dsDNA target cleavage by the TnpB RNP complex was evaluated by examining the adapter ligation at the targeted sequence in the 7N plasmid library. This was accomplished by extracting and counting all reads containing the adapter ligated at the 0–30 bp target positions next to the 7N region and identifying 10 bp of perfectly matching sequences derived from the adapter and the plasmid backbone. The analysis revealed that 1–5% of total reads have the adapter ligated at the 0–30-bp target positions. The reads exhibiting elevated frequency of adapter ligation in the target region (20–21 bp from the 7N randomized sequence) were used for 7N sequence (TAM) extraction and representation in WebLogo^40^ and position frequency matrix (PFM) formats. The Python scripts used in the cleavage position identifications and TAM characterization are provided in the GitHub repository (https://github.com/tkarvelis/Nuclease_manuscript).

### DNA substrates

Plasmid DNA substrates used in in vitro cleavage assays (pGB72-73) were obtained by cloning oligoduplexes assembled after annealing complementary synthetic oligonucleotides (Thermo Fisher Scientific) into the pSG4K5 plasmid (a gift from X. Wang, Addgene plasmid #74492) pre-cleaved with EcoRI and NheI restriction endonucleases (Thermo Fisher Scientific). The links to the plasmid sequences are provided in Supplementary Table 1 and the targeted sequences are provided in Supplementary Table 3.

Synthetic DNA substrates were 5′ end labelled by incubating 1 µM of oligonucleotide (Thermo Fisher Scientific) with 1 µl (10 U/µl) of PNK (Thermo Fisher Scientific) and ^32^P-γ-ATP (PerkinElmer) at 37 °C for 30 min in 7.5 µl of 1× reaction buffer A (Thermo Fisher Scientific). Oligoduplexes (100 nM) were obtained by combining ^32^P-labelled and unlabelled complementary oligonucleotides (1:1.5 molar ratio) followed by heating to 95 °C and slow cooling to room temperature. The sequences of the substrates are provided in Supplementary Table 2.

### DNA cleavage assays

Plasmid DNA cleavage reactions were initiated by mixing 100 nM of TnpB RNP complex with 3 nM of plasmid DNA (pGB72-73; Supplementary Table 1) in 10 mM Tris-HCl (pH 7.5 at 37 °C), 10 mM MgCl_2_, 1 mM DTT, 1 mM EDTA and 100 mM NaCl reaction buffer and incubating at 37 °C for 60 min. The reactions were quenched by adding 3× loading dye solution (0.03% bromophenol blue, 0.3% SDS and 75 mM EDTA in 30% (v/v) glycerol) and analysed by agarose gel electrophoresis and ethidium bromide staining. The linearized plasmid substrate was obtained by pre-cleaving target plasmid with NdeI endonuclease (Thermo Fisher Scientific).

Cleavage reactions with synthetic oligoduplexes (Supplementary Table 4) were initiated by combining 100 nM TnpB RNP complex with 1 nM radiolabelled substrate in 100 µl of 10 mM Tris-HCl (pH 7.5 at 37 °C), 10 mM MgCl_2_, 1 mM DTT, 1 mM EDTA and 100 mM NaCl reaction buffer at 37 °C. Aliquots of 10 μl were removed from the reaction mixture at timed intervals (0 min, 1 min, 5 min, 15 min and 60 min), quenched with 1.8× volume of a loading dye (95% (v/v) formamide, 0.01% bromophenol blue and 25 mM EDTA) and subjected to denaturing gel electrophoresis (20% polyacrylamide containing 8.5 M urea in 0.5× TBE buffer).

### Plasmid interference assay

Plasmid interference assays were performed in the *E. coli* Arctic Express (DE3) strain bearing TnpB and reRNA encoding plasmids (pGB74-76) (Supplementary Table 1). The cells were grown at 37 °C to an OD_600_ of approximately 0.5 and electroporated with 100 ng of a target-containing plasmid engineered from pSG4K5 (a gift from X. Wang, Addgene plasmid 74492) (pGB72; Supplementary Table 1). After 1 h, co-transformed cells were further diluted by serial of 10× fold dilutions and grown at 25 °C on plates containing IPTG (0.1 mM), gentamicin (10 µg/ml), carbenicillin (100 µg/ml) and kanamycin (50 µg/ml) for 44 h.

### HEK293T cell culturing and genome editing

HEK293T cells purchased from the American Type Culture Collection (catalogue number CRL-3216) were cultivated in DMEM (Gibco) supplemented with 10% FBS (Gibco), penicillin (100 U/ml) and streptomycin (100 µg/ml) (Thermo Fisher Scientific). The cell line was not further authenticated and was not tested for mycoplasma contamination. A day before transfection, the cells were plated in a 24-well plate at a density of 1.4 × 10^5^ cells per well. The transfection mixture was prepared by mixing 1 µg of plasmid encoding NLS-tagged TnpB and its reRNA (pRZ122-127; Supplementary Table 1) with 2 µl of TurboFect transfection reagent (Thermo Fisher Scientific) in 100 µl of serum-free DMEM. After 15 min of incubation at room temperature, the transfection mix was added dropwise to the cells. The cells were then grown for 72 h at 37 °C and 5% CO_2_.

### Indel characterization

Transfected HEK293T cells were trypsinized, washed and resuspended in 100 µl of PBS (approximately 6 × 10^5^ cells were collected from each well), and 10 µl of the obtained suspension was lysed using 50 µl of QuickExtract solution (Lucigen). Next, two rounds of PCR were performed: first to amplify the DNA region surrounding each target site, and second to add the sequences required for Illumina sequencing and indexing. In brief, 1–4 µl of cell lysate was used in the primary PCR with primers specific to the targeted genomic locus that were 5′ tailed with Illumina Read1 and Read2 sequences, in a final volume of 20 µl, using Hot Start Phusion Polymerase (Thermo Fisher Scientific). The thermocycler setting consisted of initial denaturation at 98 °C for 30 s, 15 cycles of 98 °C for 15 s, 56.8 °C for 15 s, 72 °C for 30 s, and final incubation at 72 °C for 5 min. The resulting amplicons were cleaned using 1.8× volume of magnetic beads (Lexogen) and eluted in 30 µl of elution buffer. Of the eluted mixture, 6 µl was used as a template for the second round of PCR, in a final volume of 30 µl, to index and add P5 and P7 adapters required for Illumina sequencing, using the Lexogen PCR Add on Kit (Lexogen) with the i7 6 nt Index Set (Lexogen). The thermocycler setting consisted of initial denaturation at 98 °C for 30 s, 15 cycles of 98 °C for 10 s, 65 °C for 20 s, 72 °C for 30 s and final incubation at 72 °C for 1 min. To ensure the purity of the PCR products, an additional cleanup with 0.9× volume of magnetic beads (Lexogen) was performed. Barcoded and purified DNA samples were quantified by Qubit 4 Fluorometer (Thermo Fisher Scientific), analysed using BioAnalyzer (Agilent), pooled in an equimolar ratio and pair-end sequenced (2 × 75 bp) using the MiniSeq High Output Reagent Kit, 150 cycles (Illumina) on a MiniSeq System (Illumina). Indels were analysed using CRISPResso2 with the following parameters: minimum of 70% homology for alignment to the amplicon sequence, quantification window of 10 bp, ignoring substitutions to avoid false positives and phred33 score of more than 10 for average read and single base pair quality^41^. The primers and target sequences are provided in Supplementary Tables 5 and 6, respectively.

### ISDra2 TnpB modelling and structure comparison

To gain insight into the structure of TnpB, we submitted separately N-terminal and C-terminal regions to the well-performing publicly available structure prediction servers trRosetta^42^, tFOLD (https://drug.ai.tencent.com) and RaptorX^43^, as established by CASP (Critical Assessment of Protein Structure Prediction). All three methods returned very similar structures for either N-terminal or C-terminal region. As expected, the C-terminal region features the RuvC domain with the inserted zinc-finger domain. The structure of the modelled N-terminal region corresponded to a β-barrel domain with the inserted three-helix bundle. A search with the trRosetta model of the N-terminal region of TnpB against the Protein Data Bank using Dali^44^ identified N-terminal lobes of other Cas12 proteins as the closest matches. The TnpB β-barrel matched a similar domain in Cas12, often referred to as the ‘Wedge’ domain, whereas the inserted helical bundle matched a part of the ‘Rec’ domain. More detailed structural comparison with available different Cas12 groups revealed that the N-terminal region of TnpB corresponds to the minimal common structural elements present in Cas12. The TnpB RuvC region also represents a streamlined version of Cas12 RuvC domain variants. Most recently, as the highly accurate AlphaFold2 structure prediction method^45^ became publicly available, we sought further investigation of the TnpB structure. AlphaFold2 produced TnpB models that were very similar to those obtained previously, suggesting that the predicted TnpB structure might be fairly accurate.

### Statistics and reproducibility

All experiments represented without replicates (for example, gels for qualitative characterization of proteins and nucleic acids) have been reproduced at least three times independently (except for the small RNA sequencing and plasmid library cleavage experiments, which were performed once).

### Reporting summary

Further information on research design is available in the Nature Research Reporting Summary linked to this paper.

## Online content

Any methods, additional references, Nature Research reporting summaries, source data, extended data, supplementary information, acknowledgements, peer review information; details of author contributions and competing interests; and statements of data and code availability are available at 10.1038/s41586-021-04058-1.

### Supplementary information

Supplementary Information This file contains Supplementary Fig. 1 (unprocessed gel images for the Main text Figs), Supplementary Fig. 2 (unprocessed gel images for the Extended Data Figs), and Supplementary Tables 1–6.

Reporting Summary

## Figures and Tables

**Extended Data Table 1:**
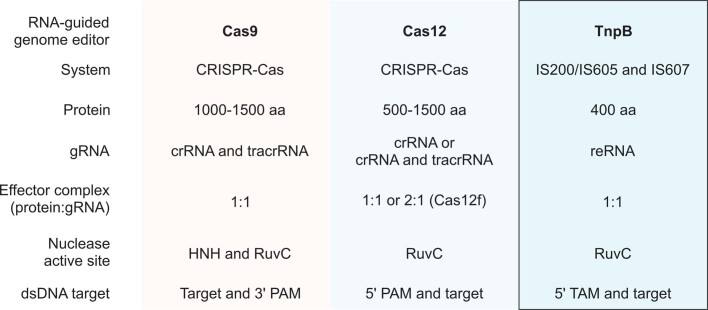
Comparison of biochemical features of RNA-guided nucleases adopted for genome editing

## Data Availability

All data are available in the paper and the supplementary material. In addition, small RNA and plasmid library cleavage sequencing data are available on the NCBI Sequence Read Archive under BioProject ID PRJNA723137. The ISfinder database was accessed at https://isfinder.biotoul.fr.

## Code availability

The Python scripts used for identification of the plasmid library cleavage positions and characterization of TAMs are available in a GitHub repository found at https://github.com/tkarvelis/Nuclease_manuscript.
