# Supplementary material for: Transposon-associated TnpB is a programmable RNA-guided DNA endonuclease
Source: Nature. 2021 Oct 7;599(7886):692–6. doi: 10.1038/s41586-021-04058-1 (PMC8612924; doi:10.1038/s41586-021-04058-1)
Supplement: Supplementary file 1 — This file contains Supplementary Fig. 1 (unprocessed gel images for the Main text Figs), Supplementary Fig. 2 (unprocessed gel images for the Extended Data Figs), and Supplementary Tables 1–6. [file 41586_2021_4058_MOESM1_ESM.pdf]

nature portfolio

https://doi.org/10.1038/s41586-021-04058-1

Supplementary information

Transposon-associated TnpB is a programmable RNA-guided DNA endonuclease

In the format provided by the authors and unedited

Supplementary Information

Transposon-associated TnpB is an RNA-programmable DNA nuclease

Tautvydas Karvelis[1,*], Gytis Druteika[1], Greta Bigelyte[1], Karolina Budre[1], Rimante Zedaveinyte[1], Arunas Silanskas[1], Darius Kazlauskas[1], Česlovas Venclovas[1], Virginijus Siksnys[1,*]

[1] Institute of Biotechnology, Life Sciences Center, Vilnius University, Vilnius, Lithuania.
[*] Correspondence to: tautvydas.karvelis@bti.vu.lt and siksnys@ibt.lt

Table of Contents

Supplementary Fig. 1 | Uncropped images for the Main text Figures 2
Supplementary Fig. 2 | Uncropped images for the Extended Data Figures. 3
Supplementary Table 1 | Plasmids used in this study 5
Supplementary Table 2 | Sequences of the TnpB protein variants used in this study 7
Supplementary Table 3 | TnpB targeted sequences in 7N library and dsDNA cleavage experiments 8
Supplementary Table 4 | Oligonucleotides used for *in vitro* cleavage assays 9
Supplementary Table 5 | Primers used in human genome editing assay 10
Supplementary Table 6 | TnpB targeted sequences in human genome editing experiment........ 11

Fig. 2e

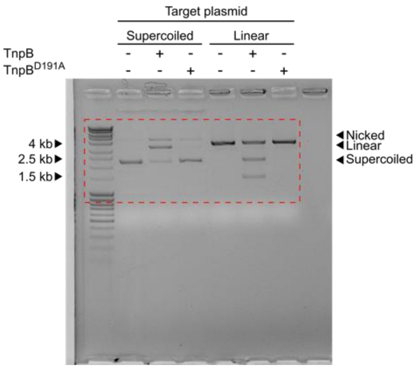

Fig. 2f

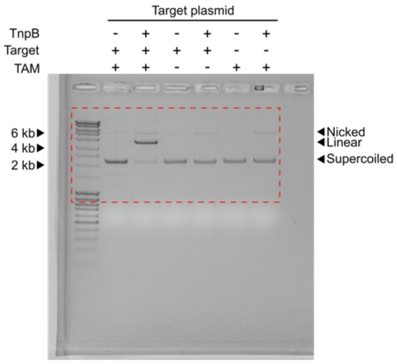

Fig. 3b

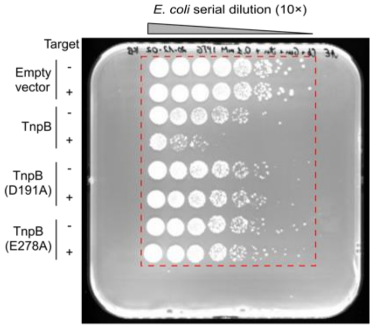

Supplementary Fig. 1 | Uncropped images for the Main Text Figures. The red rectangles indicate the cropping location.

Extended Data Fig. 1a

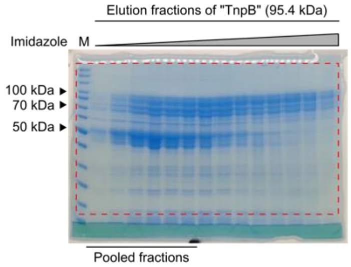

Extended Data Fig. 1b

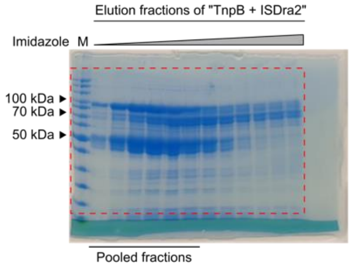

Extended Data Fig. 1c

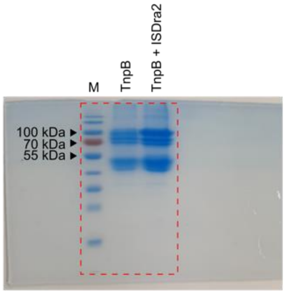

Extended Data Fig. 1d

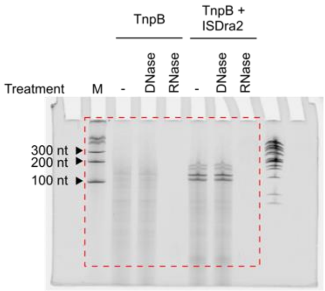

Extended Data Fig. 4b

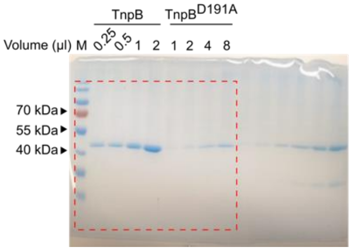

Supplementary Fig. 2 | Uncropped images for the Extended Data Figures. The red rectangles indicate the cropping location.

Extended Data Fig. 5a

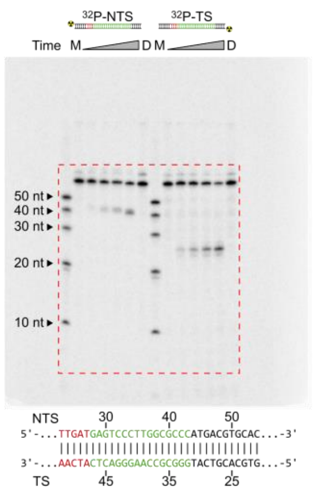

Extended Data Fig. 5b

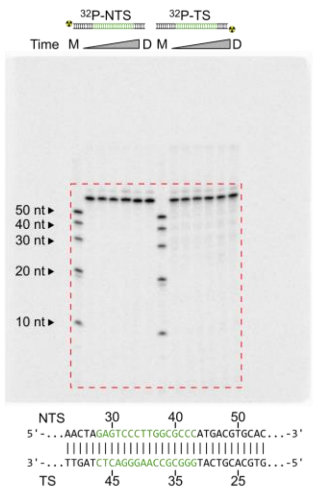

Extended Data Fig. 6a

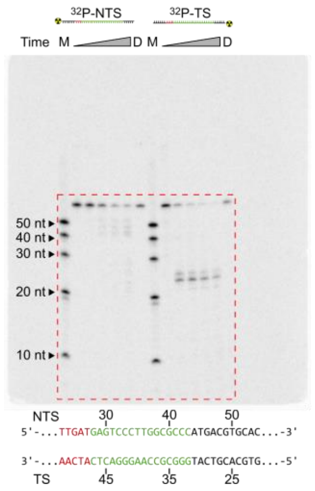

Extended Data Fig. 6b

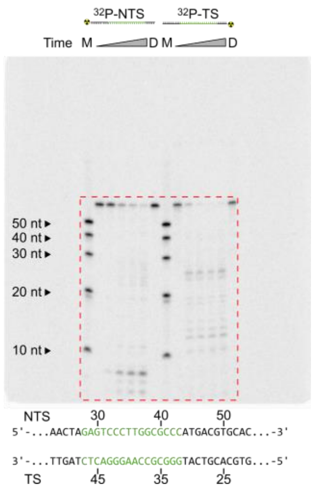

Supplementary Fig. 2 | Uncropped images for the Extended Data Figures (continued). The red rectangles indicate the cropping location.

Supplementary Table 1 | Plasmids used in this study.

Plasmid name	Description	Figures	Link
pTK120-ISDra2-TnpB	TnpB expression plasmid (TwinStrep-10×His-MBP-TEV-TnpB)	1c-d, Extended Data Fig. 1	https://benchling.com/s/seq-wOnPpT0TgQJseCavnnaR
pTWIST-ISDra2	Plasmid containing complete ISDra2 transposon system	-	https://benchling.com/s/seq-A70Ed8hYrguvmBH2Zw0A
pGD3	pTWIST-ISDra2 construct containing TnpA deletion	1c-d, Extended Data Fig. 1	https://benchling.com/s/seq-S4BsxOuWtIs1mosJHjPb
pTK151	TnpB expression plasmid (6×His-MBP-TEV-TnpB-StrepTag II)	2d, Extended Data Fig. 4	https://benchling.com/s/seq-Rd1yxYkgDNoDHL5aZRwD
pTK152	TnpB D191A expression plasmid (6×His-MBP-TEV-TnpB (D191A)-StrepTag II)	2d, Extended Data Fig. 4	https://benchling.com/s/seq-LA9lcMvtjVZl4udtfyCt
pGB71	reRNA expression plasmid	2d, Extended Data Fig. 4	https://benchling.com/s/seq-Mkw73LLrFgtP3V5fWpkQ
pGB74	TnpB and reRNA expression construct used for plasmid interference assay in *E. coli*	3a-b	https://benchling.com/s/seq-X2A1t5z7VY19ARY3ffQ7
pGB75	TnpB D191A and reRNA expression construct used for plasmid interference assay in *E. coli*	3a-b	https://benchling.com/s/seq-jEoI8nNlxvKp5XAPLpbW
pGB76	TnpB E278A and reRNA expression construct used for plasmid interference assay in *E. coli*	3a-b	https://benchling.com/s/seq-mQlFsmbzdS5WSGlbdDTu
pETDuet-1	Empty vector used for plasmid interference assay in *E. coli*	3a-b	https://benchling.com/s/seq-dWuzfZLfOTaEx2zx9nFv
pTZ57	7N plasmid library	2a-c, Extended Data Fig. 2	https://benchling.com/s/seq-5vm5J2YMrqofoxGYzNAj
pGB77	Plasmid for TnpB and reRNA expression in *E. coli* targeting 7N plasmid library (16 nt guide)	2a-c	https://benchling.com/s/seq-WQGHHuLlWZEIB960BGVm

pGB78	Plasmid for TnpB and reRNA expression in *E. coli* targeting 7N plasmid library (20 nt guide)	Extended Data Fig. 2	https://benchling.com/s/seq-WSC3bcDdfc55gYVgfopZ
pGB72	TnpB target plasmid with TAM sequence used for *in vivo* plasmid interference and *in vitro* activity experiments	2e-g, 3a-b	https://benchling.com/s/seq-SC1ze8ARFSbNhpCDwGKv
pGB73	TnpB target plasmid without TAM sequence used for *in vitro* activity experiments	2f	https://benchling.com/s/seq-b9XdVGsl4g3tUK3dVgkq
pSG4K5	Non-target plasmid used for *in vivo* plasmid interference and *in vitro* activity experiments	3a-b	https://benchling.com/s/seq-oyan25ZovFhAOLSYUEuk
pRZ122	Plasmid for TnpB and non-targeting reRNA expression in human cells	4a-b	https://benchling.com/s/seq-ZcyeLQKncrXYuOOBrxwl
pRZ123	Plasmid for TnpB and reRNA expression in human cells, targeting *EMX1-1*	4a-c	https://benchling.com/s/seq-A7J0kVZQAhu0G0dvocYA
pRZ124	Plasmid for TnpB and reRNA expression in human cells, targeting *EMX1-2*	4a-b	https://benchling.com/s/seq-5Myy239GbPkHFjIiZSJP
pRZ125	Plasmid for TnpB and reRNA expression in human cells, targeting *HPRT1*	4a-b	https://benchling.com/s/seq-gbyVUxBIWAnaVmYThZtV
pRZ126	Plasmid for TnpB and reRNA expression in human cells, targeting *AGBL1-1*	4a-b	https://benchling.com/s/seq-vaf08y4lbzDTSIgCDF3C
pRZ127	Plasmid for TnpB and reRNA expression in human cells, targeting *AGBL1-2*	4a-b	https://benchling.com/s/seq-yZ3derrT6fAOPmDJNC1o

Supplementary Table 2 | Sequences of the TnpB protein variants used in this study.

TnpB

MIRNKAFVVRLYPNAAQTELINRTLGSARFVYNHFLARRIAAYKESGKGLTYGQTSSELTLLKQAEETSWLSEVDKFAL
QNSLKNLETAYKNFFRTVKQSGKKVGFPRFRKKRTGESYRTQFTNNNIQIGEGRLKLPKLGWVKTKGQQDIQGKILNV
TVRRIHEGHYEASVLCEVEIPYLPAAPKFAAGVDVGIKDFAIVTDGVRFKHEQNPKYYRSTLKRLRKAQQTLSRRKKGS
ARYGKAKTKLARIHKRIVNKRQDFLHKLTTSLVREYEIIGTEHLKPDNMRKNRRLALSISDAGWGEFIRQLEYKAAWYG
RLVSKVSPYFPSSQLCHDCGFKNPEVKNLAVRTWTCPNCGETHDRDENAALNIRREALVAAGISDTLNAHGGYVRPA
SAGNGLRSENHATLVV*

TwinStrep-10×His-MBP-TEV (cleavage site)-TnpB

MGGSAWSHPQFEKGGGSGGGSGGSAWSHPQFEKGSMGGSHHHHHHHHHHGMASMKIEEGKLVIWINGDKGYNG
LAEVGKKFEKDTGIKVTVEHPDKLEEKFPQVAATGDGPDIIFWAHDRFGGYAQSGLLAEITPDKAFQDKLYPFTWDAV
RYNGKLIAYPIAVEALSLIYNKDLLPNPPKTWEEIPALDKELKAKGKSALMFNLQEPYFTWPLIAADGGYAFKYENGKYD
IKDVGVDNAGAKAGLTFLVDLIKNKHMNADTDYSIAEAAFNKGETAMTINGPWAWSNIDTSKVNYGVTVLPTFKGQPS
KPFVGVLSAGINAASPNKELAKEFLENYLLTDEGLEAVNKDKPLGAVALKSYEEELAKDPRIAATMENAQKGEIMPNIP
QMSAFWYAVRTAVINAASGRQTVDEALKDAQTNSSSNNNNNNNNNNLGIEENLYFQSNAGGGGMIRNKAFVVRLYP
NAAQTELINRTLGSARFVYNHFLARRIAAYKESGKGLTYGQTSSELTLLKQAEETSWLSEVDKFALQNSLKNLETAYKN
FFRTVKQSGKKVGFPRFRKKRTGESYRTQFTNNNIQIGEGRLKLPKLGWVKTKGQQDIQGKILNVTVRRIHEGHYEAS
VLCEVEIPYLPAAPKFAAGVDVGIKDFAIVTDGVRFKHEQNPKYYRSTLKRLRKAQQTLSRRKKGSARYGKAKTKLARI
HKRIVNKRQDFLHKLTTSLVREYEIIGTEHLKPDNMRKNRRLALSISDAGWGEFIRQLEYKAAWYGRLVSKVSPYFPSS
QLCHDCGFKNPEVKNLAVRTWTCPNCGETHDRDENAALNIRREALVAAGISDTLNAHGGYVRPASAGNGLRSENHA
TLVV*

6×His-MBP-TEV (cleavage site)-TnpB-StrepTag II

MGGSHHHHHHGMASMKIEEGKLVIWINGDKGYNGLAEVGKKFEKDTGIKVTVEHPDKLEEKFPQVAATGDGPDIIFW
AHDRFGGYAQSGLLAEITPDKAFQDKLYPFTWDAVRYNGKLIAYPIAVEALSLIYNKDLLPNPPKTWEEIPALDKELKAK
GKSALMFNLQEPYFTWPLIAADGGYAFKYENGKYDIKDVGVDNAGAKAGLTFLVDLIKNKHMNADTDYSIAEAAFNKG
ETAMTINGPWAWSNIDTSKVNYGVTVLPTFKGQPSKPFVGVLSAGINAASPNKELAKEFLENYLLTDEGLEAVNKDKP
LGAVALKSYEEELAKDPRIAATMENAQKGEIMPNIPQMSAFWYAVRTAVINAASGRQTVDEALKDAQTNSSSNNNNN
NNNNNLGIEENLYFQSNAGGGGMIRNKAFVVRLYPNAAQTELINRTLGSARFVYNHFLARRIAAYKESGKGLTYGQTS
SELTLLKQAEETSWLSEVDKFALQNSLKNLETAYKNFFRTVKQSGKKVGFPRFRKKRTGESYRTQFTNNNIQIGEGRL
KLPKLGWVKTKGQQDIQGKILNVTVRRIHEGHYEASVLCEVEIPYLPAAPKFAAGVDVGIKDFAIVTDGVRFKHEQNPK
YYRSTLKRLRKAQQTLSRRKKGSARYGKAKTKLARIHKRIVNKRQDFLHKLTTSLVREYEIIGTEHLKPDNMRKNRRLA
LSISDAGWGEFIRQLEYKAAWYGRLVSKVSPYFPSSQLCHDCGFKNPEVKNLAVRTWTCPNCGETHDRDENAALNIR
REALVAAGISDTLNAHGGYVRPASAGNGLRSENHATLVVGSGWSHPQFEK*

6×His-MBP-TEV (cleavage site)-TnpB (D191A)-StrepTag II

MGGSHHHHHHGMASMKIEEGKLVIWINGDKGYNGLAEVGKKFEKDTGIKVTVEHPDKLEEKFPQVAATGDGPDIIFW
AHDRFGGYAQSGLLAEITPDKAFQDKLYPFTWDAVRYNGKLIAYPIAVEALSLIYNKDLLPNPPKTWEEIPALDKELKAK
GKSALMFNLQEPYFTWPLIAADGGYAFKYENGKYDIKDVGVDNAGAKAGLTFLVDLIKNKHMNADTDYSIAEAAFNKG
ETAMTINGPWAWSNIDTSKVNYGVTVLPTFKGQPSKPFVGVLSAGINAASPNKELAKEFLENYLLTDEGLEAVNKDKP
LGAVALKSYEEELAKDPRIAATMENAQKGEIMPNIPQMSAFWYAVRTAVINAASGRQTVDEALKDAQTNSSSNNNNN
NNNNNLGIEENLYFQSNAGGGGMIRNKAFVVRLYPNAAQTELINRTLGSARFVYNHFLARRIAAYKESGKGLTYGQTS
SELTLLKQAEETSWLSEVDKFALQNSLKNLETAYKNFFRTVKQSGKKVGFPRFRKKRTGESYRTQFTNNNIQIGEGRL
KLPKLGWVKTKGQQDIQGKILNVTVRRIHEGHYEASVLCEVEIPYLPAAPKFAAGVAVGIKDFAIVTDGVRFKHEQNPK
YYRSTLKRLRKAQQTLSRRKKGSARYGKAKTKLARIHKRIVNKRQDFLHKLTTSLVREYEIIGTEHLKPDNMRKNRRLA
LSISDAGWGEFIRQLEYKAAWYGRLVSKVSPYFPSSQLCHDCGFKNPEVKNLAVRTWTCPNCGETHDRDENAALNIR
REALVAAGISDTLNAHGGYVRPASAGNGLRSENHATLVVGSGWSHPQFEK*

Supplementary Table 3 | TnpB targeted sequences in 7N library and dsDNA cleavage experiments.

**Target plasmid**	**Sequence 5'→3' (TAM, Target)**	**Figures**
pTZ57	NNNNNNNAGTTGACCCAACGTCG	2a-c
pTZ57	NNNNNNNAGTTGACCCAACGTCGCCGG	Extended Data Fig. 2
pGB72	TTGATGAGTCCCTTGGCGCCC	2e-g, 3a-b
pGB73	AACTAGAGTCCCTTGGCGCCC	2f

Supplementary Table 4 | Oligonucleotides used for *in vitro* cleavage assays.

**Description**	**Sequence 5’→3’ (TAM, Target)**	**Figures**
Target (NTS)	GCACCTTACTGCAAGGTAGCGCTTGATGAGTCCCTTGGCGCCCATG<br>ACGTGCACAATCTAGATGCATCAGCTGC	Extended Data Fig. 5a, Extended Data Fig. 6a
Target (TS)	GCAGCTGATGCATCTAGATTGTGCACGTCATGGGCGCCAAGGGACT<br>CATCAAGCGCTACCTTGCAGTAAGGTGC	Extended Data Fig. 5a, Extended Data Fig. 6a
Target (NTS)	GCACCTTACTGCAAGGTAGCGGAACTAGAGTCCCTTGGCGCCCATG<br>ACGTGCACAATCTAGATGCATCAGCTGC	Extended Data Fig. 5b, Extended Data Fig. 6b
Target (TS)	GCAGCTGATGCATCTAGATTGTGCACGTCATGGGCGCCAAGGGACT<br>CTAGTTCCGCTACCTTGCAGTAAGGTGC	Extended Data Fig. 5b, Extended Data Fig. 6b
Marker	GCACCTTACTGCAAGGTAGCGTATTTAAGTTGACCCAACGTCGCCG<br>GCGT	Extended Data Fig. 5, Extended Data Fig. 6
	GCACCTTACTGCAAGGTAGCGTATTTAAGTTGACCCAACG	
	GCACCTTACTGCAAGGTAGCGTATTTAAGT	
	GCACCTTACTGCAAGGTAGC	
	GCACCTTACT	

Supplementary Table 5 | Primers used in human genome editing assay. Blue and red fonts represent Read1 and Read2 sequences, respectively, required for Illumina sequencing. Black font indicates the sequence used for amplification of the genomic locus.

**Description**	**Sequence (5'→3')**
Primary PCR *EMX1-1* (forward)	ACACTCTTTCCCTACACGACGCTCTTCCGATCTGTACAAACGGCAGAAGCTGG
Primary PCR *EMX1-1* (reverse)	GTGACTGGAGTTCAGACGTGTGCTCTTCCGATCTCTTCGTGGCAATGCGCC
Primary PCR *EMX1-2* (forward)	ACACTCTTTCCCTACACGACGCTCTTCCGATCTCTCCGTGTCTCCAATCTCCC
Primary PCR *EMX1-2* (reverse)	GTGACTGGAGTTCAGACGTGTGCTCTTCCGATCTCCCATAGGGAAGGGGGACAC
Primary PCR *HPRT1* (forward)	ACACTCTTTCCCTACACGACGCTCTTCCGATCTGTGATGATGAACCAGGTTATGAC
Primary PCR *HPRT1* (reverse)	GTGACTGGAGTTCAGACGTGTGCTCTTCCGATCTGTCCATGAGGAATAAACACCC
Primary PCR *AGBL1-1* (forward)	ACACTCTTTCCCTACACGACGCTCTTCCGATCTCATGTTCCTCACAAAGAGAGTC
Primary PCR *AGBL1-1* (reverse)	GTGACTGGAGTTCAGACGTGTGCTCTTCCGATCTCAGTTGATAATGAATGGCTACTC
Primary PCR *AGBL1-2* (forward)	ACACTCTTTCCCTACACGACGCTCTTCCGATCTATTGTTGGCTCAAACACCAG
Primary PCR *AGBL1-2* (reverse)	GTGACTGGAGTTCAGACGTGTGCTCTTCCGATCTACTCTATGGTGAATTGTCAAGC

Supplementary Table 6 | TnpB targeted sequences in human genome editing experiment.

**Target**	**Target sequence (5'→3')**
Non-targeting (NT)	AGTTGACCCAACGTCGCCGG
EMX1-1	GTGATGGGAGCCCTTCTTCT
EMX1-2	GCATTTCTGTTTTAATTTAT
HPRT1	TTATTTTGCATACCTAATCA
AGBL1-1	TGTTGGCTCAAACACCAGAT
AGBL1-2	AATGAATGGCTACTCTAACC
