# Supplementary material for: Transposon-associated TnpB is a programmable RNA-guided DNA endonuclease
Source: Nature. 2021 Oct 7;599(7886):692–6. doi: 10.1038/s41586-021-04058-1 (PMC8612924; doi:10.1038/s41586-021-04058-1)
Supplement: Supplementary file 2 — Reporting Summary [file 41586_2021_4058_MOESM2_ESM.pdf]

nature portfolio

Corresponding author(s): Tautvydas Karvelis and Virginijus Siksnys

Last updated by author(s): Sep 20, 2021

Reporting Summary

Nature Portfolio wishes to improve the reproducibility of the work that we publish. This form provides structure for consistency and transparency in reporting. For further information on Nature Portfolio policies, see our Editorial Policies and the Editorial Policy Checklist.

Statistics

For all statistical analyses, confirm that the following items are present in the figure legend, table legend, main text, or Methods section.

n/a	Confirmed	
☐	☒	The exact sample size (*n*) for each experimental group/condition, given as a discrete number and unit of measurement
☐	☒	A statement on whether measurements were taken from distinct samples or whether the same sample was measured repeatedly
☒	☐	The statistical test(s) used AND whether they are one- or two-sided<br>*Only common tests should be described solely by name; describe more complex techniques in the Methods section.*
☒	☐	A description of all covariates tested
☒	☐	A description of any assumptions or corrections, such as tests of normality and adjustment for multiple comparisons
☐	☒	A full description of the statistical parameters including central tendency (e.g. means) or other basic estimates (e.g. regression coefficient) AND variation (e.g. standard deviation) or associated estimates of uncertainty (e.g. confidence intervals)
☒	☐	For null hypothesis testing, the test statistic (e.g. *F*, *t*, *r*) with confidence intervals, effect sizes, degrees of freedom and *P* value noted<br>*Give P values as exact values whenever suitable.*
☒	☐	For Bayesian analysis, information on the choice of priors and Markov chain Monte Carlo settings
☒	☐	For hierarchical and complex designs, identification of the appropriate level for tests and full reporting of outcomes
☒	☐	Estimates of effect sizes (e.g. Cohen's *d*, Pearson's *r*), indicating how they were calculated

Our web collection on statistics for biologists contains articles on many of the points above.

Software and code

Policy information about availability of computer code

Data collection	Next-generation sequencing data was obtained using Illumina MiSeq and MiniSeq platforms
Data analysis	CRISPResso2 (v2.0.20b) (https://crispresso.pinellolab.partners.org/), AcquireMP (v2.3.0). The Python scripts used for plasmid library cleavage position identification and TAM characterization are provided at GitHub repository (https://github.com/tkarvelis/Nuclease_manuscript).

For manuscripts utilizing custom algorithms or software that are central to the research but not yet described in published literature, software must be made available to editors and reviewers. We strongly encourage code deposition in a community repository (e.g. GitHub). See the Nature Portfolio guidelines for submitting code & software for further information.

Data

Policy information about availability of data

All manuscripts must include a data availability statement. This statement should provide the following information, where applicable:

- Accession codes, unique identifiers, or web links for publicly available datasets
- A description of any restrictions on data availability
- For clinical datasets or third party data, please ensure that the statement adheres to our policy

All data are available in the manuscript and the supplementary material. Additionally, sRNA and plasmid library cleavage sequencing data are available on the NCBI Sequence Read Archive under BioProject ID PRJNA723137. ISfinder database was accessed at https://isfinder.biotoul.fr.

Field-specific reporting

Please select the one below that is the best fit for your research. If you are not sure, read the appropriate sections before making your selection.

☒ Life sciences ☐ Behavioural & social sciences ☐ Ecological, evolutionary & environmental sciences

For a reference copy of the document with all sections, see nature.com/documents/nr-reporting-summary-flat.pdf

Life sciences study design

All studies must disclose on these points even when the disclosure is negative.

Sample size	Sample size (n=3) was based on the work and experience of other groups in the field who generate reproducible results in the similar design experiments with the cells (no statistical methods were used to determine the sample size).
Data exclusions	No data was excluded from the analysis.
Replication	Reproducibility was ensured by repeating most of the experiments independently at least 3 times.
Randomization	Samples were not randomized as it is not applicable for the current design of the study.
Blinding	Investigators were not blinded as it is not applicable for the current design of the study.

Reporting for specific materials, systems and methods

We require information from authors about some types of materials, experimental systems and methods used in many studies. Here, indicate whether each material, system or method listed is relevant to your study. If you are not sure if a list item applies to your research, read the appropriate section before selecting a response.

Materials & experimental systems

n/a	Involved in the study	
☒	☐	Antibodies
☐	☒	Eukaryotic cell lines
☒	☐	Palaeontology and archaeology
☒	☐	Animals and other organisms
☒	☐	Human research participants
☒	☐	Clinical data
☒	☐	Dual use research of concern

Methods

n/a	Involved in the study	
☒	☐	ChIP-seq
☒	☐	Flow cytometry
☒	☐	MRI-based neuroimaging

Eukaryotic cell lines

Policy information about cell lines

Cell line source(s)	HEK293T cell line was obtained from ATCC (CRL-3216™).
Authentication	HEK293T cell line was not authenticated.
Mycoplasma contamination	HEK293T cell line was not tested for mycoplasma contamination.
Commonly misidentified lines (See ICLAC register)	HEK293T cell line is not listed in the ICLAC register of misidentified cell lines.
